# The geriatric nutritional risk index predicts complications after nephrectomy for renal cancer

**DOI:** 10.1590/S1677-5538.IBJU.2022.0380

**Published:** 2022-10-03

**Authors:** Carlos Riveros, Victor Chalfant, Soroush Bazargani, Mark Bandyk, Kethandapatti Chakravarthy Balaji

**Affiliations:** 1 University of Florida Department of Urology Jacksonville FL USA Department of Urology, University of Florida, Jacksonville, FL 32209, USA

**Keywords:** Kidney Neoplasms, Nephrectomy, Nutrition Assessment

## Abstract

**Purpose:**

We examined if malnutrition, as defined by the Geriatric Nutritional Risk Index (GNRI), is independently associated with 30-day postoperative complications in patients undergoing nephrectomy for the treatment of renal cancer.

**Materials and methods:**

Using the American College of Surgeons National Surgical Quality Improvement Program database from 2006-2019, we identified patients ≥65 years old who underwent nephrectomy for renal cancer. The following formula for GNRI was used to define preoperative nutritional status: 1.489 x serum albumin (g/L) + 41.7 x (current body weight [kg]/ ideal body weight [kg]). Based on the GNRI, patients were classified as having no (> 98), moderate (92-98), or severe malnutrition (< 92). After adjusting for potential confounders, multivariable logistic regression analyses were performed to assess the association between GNRI and 30-day postoperative complications. Odds ratios (OR) with 95% confidence intervals (CI) were reported.

**Results:**

A total of 7,683 patients were identified, of which 1,241 (16.2%) and 872 (11.3%) had moderate and severe malnutrition, respectively. Compared to normal nutrition, moderate and severe malnutrition were significantly associated with a greater odds of superficial surgical site infection, progressive renal insufficiency, readmission, extended length of stay, and non-home discharge. Severe malnutrition was also associated with urinary tract infection (OR 2.10, 95% CI 1.31-3.35) and septic shock (OR 2.93, 95% CI 1.21-7.07).

**Conclusion:**

Malnutrition, as defined by a GNRI ≤ 98, is an independent predictor of 30-day complications following nephrectomy. The GNRI could be used to counsel elderly patients with renal cancer prior to nephrectomy.

## INTRODUCTION

In 2020, more than 430,000 people were diagnosed with renal cancer and approximately 180,000 people died from this disease worldwide (1). The incidence of kidney cancer is strongly related to age. Approximately 50% of new diagnoses are in elderly patients (≥ 65 years of age) (2). Nephrectomy remains the gold standard treatment in most cases (3). Overall complication rates for nephrectomy range between 10 and 30% depending on the surgical approach and extent (4).

Perioperative malnutrition is a known independent predictor of poor postoperative outcomes in general surgery, especially among the elderly (5). Malnutrition can be as high as 70% in elderly oncology patients (6–8). Screening tools can aid in identifying nutritional status among older patients undergoing oncological surgery, but the abundance of questionnaires and calculators has impeded widespread implementation of perioperative nutritional assessment in clinical practice (5, 9, 10).

Among the many tools available to assess preoperative nutritional status in the elderly, the Geriatric Nutritional Risk Index (GNRI) is simple to calculate, as it utilizes changes in height and weight, in addition to serum albumin (11). While the GNRI was first developed for predicting nutrition-related risk of morbidity and mortality among hospitalized geriatric patients, it has also been found to be an independent predictor of outcomes among elderly patients with cancer (11–13). In renal cancer, the GNRI has been shown to be an independent predictor of overall, cancer-specific, and recurrence-free survival (14, 15). However, the role of GNRI in predicting short-term outcomes after nephrectomy has not been thoroughly evaluated (16). We postulated that malnutrition, as defined by GNRI, may be independently associated with worse 30-day outcomes following nephrectomy for renal cancer. To test this hypothesis, we performed a retrospective cohort study of patients undergoing nephrectomy for renal cancer, evaluating the association between GNRI and 30-day postoperative outcomes.

## MATERIALS AND METHODS

### Study design

After receiving exempt status from our Institutional Review Board (#202101589), we queried the American College of Surgeons National Surgical Quality Improvement Program (ACS-NSQIP) database. Using data from 2006 to 2019, we identified patients ≥ 65 years old with a diagnosis of renal cancer (International Coding of Diseases, Ninth Revision [ICD-9] codes 189.x and ICD-10 codes C64.x) undergoing radical (Current Procedural Terminology [CPT] codes 50220, 50225, 50230, 50545, 50546) and partial nephrectomy (CPT codes 50240 and 50543). To account for the half-life of albumin, only patients who had serum albumin levels measured within 30 days prior to surgery were included. Patients with an American Society of Anesthesiologists (ASA) classification score of 5, ascites, preoperative sepsis, or ventilator dependence at the time of surgery were excluded. We also excluded emergency cases and patients with missing data, except for patients with missing race or ethnicity data.

### Variables

Patient preoperative profiles included age, sex, body mass index (BMI), functional status, ASA classification, 5-item modified frailty index (5i-mFI), smoking status, comorbidities (diabetes mellitus [DM], hypertension [HT], congestive heart failure [CHF], severe chronic obstructive pulmonary disease [COPD], dyspnea, disseminated cancer, > 10% body weight loss in the 6 months preceding surgery, chronic steroid use, bleeding disorder, preoperative dialysis, acute renal failure), and preoperative laboratory values (serum creatinine, hematocrit, white blood cell count, and platelet count). All preoperative laboratory values included were measured within 30 days prior to surgery. According to NSQIP, bleeding disorder is defined as “increased risk of bleeding due to an underlying hematological disorder or chronic anticoagulation” (17). Operative data included nephrectomy extent (partial or radical), surgical approach (open or minimally invasive [MI]), wound classification (I – clean, II clean/contaminated, III – contaminated, IV – dirty/infected), and operative time. CPT codes 50220, 50225, 50230, and 50240 were classified as open while CPT codes 50545, 50546, and 50543 were classified as MI. ASA classification was dichotomized into two categories: ≤ Class 2 and > Class 2. The 5i-mFI has been observed to predict postoperative complications following urologic surgery (18, 19). It is calculated based on the presence of the following: DM, HT requiring medication, CHF, respiratory disease (COPD or pneumonia), and partial/total dependence prior to surgery (20).

### Nutritional Assessment

According to previously published literature, malnutrition was defined based on BMI < 18.5 kg/m², serum albumin < 3.5 g/dL, and > 10% body weight loss in the last 6 months (5). Nutritional status according to the GNRI was calculated using the following variables: weight, height, and preoperative serum albumin. GNRI was calculated as: (1.489 x serum albumin [g/L]) + (41.7 × [current body weight (CBW)/ ideal body weight (IBW)]) (11). IBW was calculated as: (height x height) x 22, and it was capped at 1 if it exceeded the CBW (11, 21). Following a similar classification to that used by Bouillane et al., we categorized patients into three groups: severe malnutrition (GNRI < 92), moderate malnutrition (GNRI 92 - 98), and normal nutritional status (GNRI > 98) (11).

### Endpoints

The primary outcome was rate of any NSQIP complication, defined as: cardiac arrest requiring cardiopulmonary resuscitation, myocardial infarction, death, pneumonia, unplanned intubation, failure to wean from ventilator, pulmonary embolism (PE), deep venous thrombosis (DVT) requiring therapy, stroke, intraoperative or postoperative transfusion, sepsis or septic shock, acute renal failure, progressive renal insufficiency, urinary tract infection (UTI), wound dehiscence, or surgical site infection (SSI) [superficial, deep incisional, or organ/space]. Secondary endpoints were: major complications (defined as Clavien-Dindo [CD] III and IV), extended length of stay (LOS), readmission, reoperation, and non-home discharge (22).

Major complications included cardiac arrest requiring cardiopulmonary resuscitation, myocardial infarction, unplanned intubation, failure to wean from ventilator, PE, stroke, sepsis or septic shock, acute renal failure, deep incisional SSI, and organ/space SSI. Extended LOS was defined as hospital stay > 75th percentile (partial nephrectomy > 4 days and radical nephrectomy > 6 days). Non-home discharge was defined as any discharge destination that was not ‘home’ or ‘facility which was home’. Complications were also reported and analyzed individually.

#### Statistical analysis

Categorical variables were reported as frequencies (%) while continuous variables were reported as median with interquartile range (IQR). Chi-square and Kruskal-Wallis tests were used where appropriate for categorical and continuous variables. Multivariable logistic regression analyses (MLRA) were used to determine if GNRI was an independent predictor of 30-day complications following nephrectomy. Besides GNRI, the other confounders used in the models were age (continuous), sex (male vs. female), race (Caucasian vs. African American, Other, or Unknown), ASA classification (< 2 vs. ≥ 3), BMI (continuous), 5i-mFI (0 vs. 1, 2, or ≥ 3), bleeding disorder (no vs. yes), dyspnea (no vs. yes), steroid/immunosuppressant for a chronic condition (no vs. yes), smoker (no vs. yes), disseminated cancer (no vs. yes); preoperative blood transfusion (no vs. yes), acute renal failure (no vs. yes), dialysis requirement (no vs. yes), hematocrit (continuous), creatinine (continuous), hematocrit (continuous), white blood cell count (continuous), platelet count (continuous), nephrectomy type (radical vs. partial), surgical approach (open vs. MI), wound classification (I vs. II, or III, or IV), and operative time (continuous).

Two sets of MLRA models were executed: one with GNRI as a categorical variable (GNRI > 98 as the reference group vs. GNRI 92 - 98 or GNRI < 92), and the other one with GNRI as a decreasing continuous variable. Results of the analyses were reported as odds ratios (OR) with their corresponding 95% confidence intervals (95% CI). The variance inflation factor (VIF) was examined to check for multicollinearity among the models. Variables with a VIF ≥ 2.5 were considered significantly multicollinear and were eliminated from the models. Because patients with disseminated cancer were included in the original cohort, we performed a sensitivity analysis without patients undergoing cytoreductive nephrectomy to investigate whether statistically significant differences could be attributed to metastatic disease. All statistical analyses were performed using the EZR plugin for R (23). Statistical significance was set at p-value < 0.05, and all tests were two-tailed.

## RESULTS

**Figure 1:**
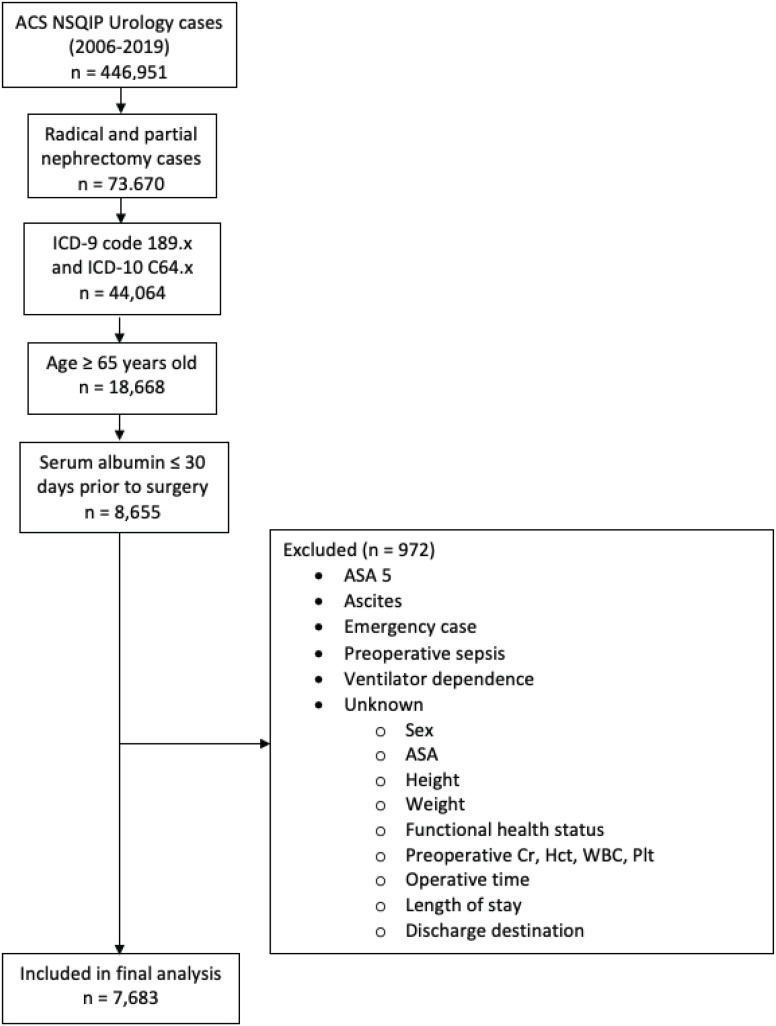
Flowchart for patient selection.

**Figure 2:**
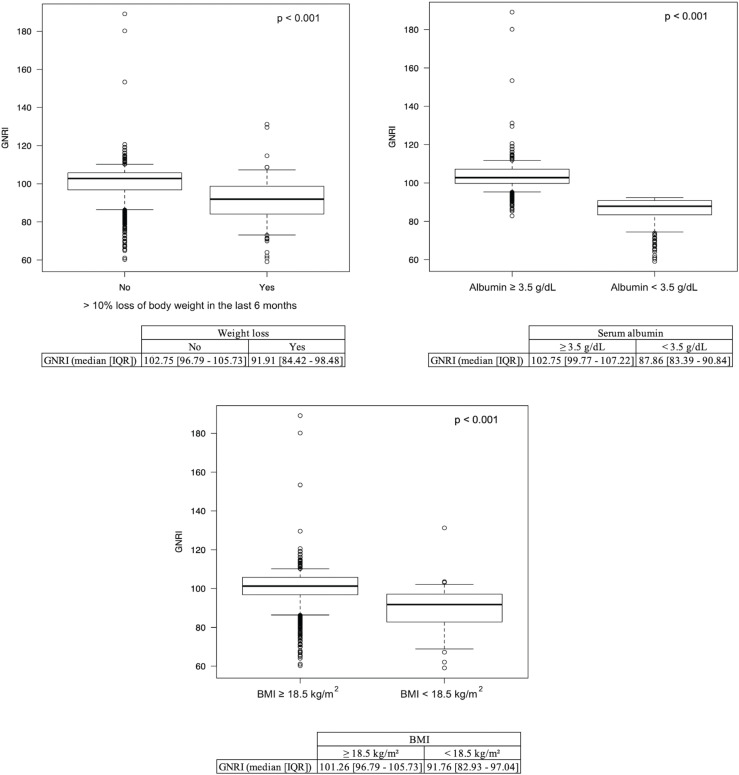
Boxplots for GNRI score compared to low albumin (< 3.5 g/dL), low BMI (< 18.5 kg/m²), and > 10% body weight loss in the last 6 months prior to nephrectomy.

A total of 7,683 cases met the inclusion criteria with 241 (16.2%) and 872 (11.3%) identified as having moderate (GNRI 92 - 98) or severe malnutrition (GNRI < 92) respectively (Figure-1). The demographic and operative profiles of all patients were summarized (Table-1). The number of patients meeting the definition of malnutrition according to BMI (< 18.5 kg/m²) and serum albumin (< 3.5 g/dL) was 58 (0.7%) and 1,039 (13.2%), respectively. Additionally, 184 (2.3%) patients were classified as having lost more than 10% of their body weight within 6 months prior to surgery. Overall, patients with a BMI < 18.5 kg/m², serum albumin < 3.5 g/dL, and > 10% body weight loss had significantly lower GNRI scores compared to their counterparts (Figure-2).

**Table 1:** Patient Demographics and Surgical Procedure Characteristics.

Variables		All patients (n=7863)	GNRI <92 (n=872)	GNRI 92-98 (n=1241)	GNRI >98 (n=5750)
**Sex**					
	Male	4948 (62.9%)	511 (58.6%)	754 (60.8%)	3683 (64.1%)
	Female	2915 (37.1%)	361 (41.4%)	487 (39.2%)	2067 (35.9%)
**Race**					
	Caucasian	6233 (79.3%)	672 (77.1%)	952 (76.7%)	4609 (80.2%)
	African American	609 (7.7%)	81 (9.3%)	118 (9.5%)	410 (7.1%)
	Other	264 (3.4%)	35 (4.0%)	37 (3.0%)	192 (3.3%)
	Unknown	757 (9.6%)	84 (9.6%)	134 (10.8%)	539 (9.4%)
**Hispanic ethnicity**					
	Yes	437 (5.6%)	48 (5.5%)	69 (5.6%)	320 (5.6%)
	No	6730 (85.6%)	721 (82.7%)	1051 (84.7%)	4958 (86.2%)
	Unknown	696 (8.9%)	103 (11.8%)	121 (9.8%)	472 (8.2%)
Age (years)		71.00 [68.00 - 76.00]	72.00 [68.00 - 77.00]	72.00 [68.00 - 77.00]	71.00 [67.00 - 75.00]
BMI (kg/m²)		29.01 [25.69 - 33.17]	27.08 [23.51 - 32.14]	28.89 [25.11 - 33.73]	29.25 [26.11 - 33.20]
Weight loss (%)		184 (2.3%)	92 (10.6%)	38 (3.1%)	54 (0.9%)
**ASA**					
	≤2	1934 (24.6%)	128 (14.7%)	230 (18.5%)	1576 (27.4%)
	>2	5929 (75.4%)	744 (85.3%)	1011 (81.5%)	4174 (72.6%)
**Functional Status**					
	Independent	7715 (98.1%)	819 (93.9%)	1204 (97.0%)	5692 (99.0%)
	Dependent	148 (1.9%)	53 (6.1%)	37 (3.0%)	58 (1.0%)
**5i-MFI**					
	0	1471 (18.7%)	171 (19.6%)	219 (17.6%)	1081 (18.8%)
	1	4091 (52.0%)	406 (46.6%)	628 (50.6%)	3057 (53.2%)
	2	2090 (26.6%)	243 (27.9%)	355 (28.6%)	1492 (25.9%)
	≥3	211 (2.7%)	52 (6.0%)	39 (3.1%)	120 (2.1%)
Current smoker		856 (10.9%)	108 (12.4%)	152 (12.2%)	596 (10.4%)
Albumin (g/dL)		4.10 [3.70 - 4.30]	3.10 [2.75 - 3.20]	3.60 [3.50 - 3.70]	4.20 [4.00 - 4.40]
Creatinine (mg/dL)		1.01 [0.84 - 1.30]	1.10 [0.85 - 1.41]	1.04 [0.87 - 1.33]	1.00 [0.83 - 1.25]
Hematocrit (%)		40.00 [36.20 - 43.10]	33.40 [29.60 - 37.50]	38.00 [34.20 - 41.90]	41.00 [37.90 - 43.70]
Platelets (mm³)		230.00 [189.00 - 282.00]	271.50 [210.75 - 368.25]	234.00 [189.00 - 293.00]	225.00 [187.00 - 272.00]
White blood cells (mm³)		7.10 [5.90 - 8.60]	7.90 [6.33 - 9.80]	7.30 [6.00 - 8.70]	7.00 [5.80 - 8.40]
Diabetes mellitus		2129 (27.1%)	243 (27.9%)	366 (29.5%)	1520 (26.4%)
Steroid use		290 (3.7%)	34 (3.9%)	49 (3.9%)	207 (3.6%)
Congestive heart failure		79 (1.0%)	29 (3.3%)	14 (1.1%)	36 (0.6%)
Hypertension		6073 (77.2%)	654 (75.0%)	947 (76.3%)	4472 (77.8%)
COPD		481 (6.1%)	70 (8.0%)	95 (7.7%)	316 (5.5%)
Dyspnea at rest/exertion		717 (9.1%)	126 (14.4%)	151 (12.2%)	440 (7.7%)
Preoperative blood transfusion		79 (1.0%)	48 (5.5%)	12 (1.0%)	19 (0.3%)
Bleeding disorder		261 (3.3%)	57 (6.5%)	57 (4.6%)	147 (2.6%)
Disseminated cancer		513 (6.5%)	143 (16.4%)	109 (8.8%)	261 (4.5%)
Preoperative AKI		33 (0.4%)	9 (1.0%)	6 (0.5%)	18 (0.3%)
Dialysis		210 (2.7%)	67 (7.7%)	50 (4.0%)	93 (1.6%)
**Nephrectomy type**					
	Partial	4661 (59.3%)	3076 (53.5%)	859 (69.2%)	726 (83.3%)
	Radical	3202 (40.7%)	2674 (46.5%)	382 (30.8%)	146 (16.7%)
**Surgical approach**					
	Robotic/Laparoscopic	2540 (32.3%)	414 (47.5%)	446 (35.9%)	1680 (29.2%)
	Open	5323 (67.7%)	458 (52.5%)	795 (64.1%)	4070 (70.8%)
**Wound classification**					
	I - Clean	1653 (21.0%)	199 (22.8%)	256 (20.6%)	1198 (20.8%)
	II - Clean/Contaminated	6126 (77.9%)	655 (75.1%)	957 (77.1%)	4514 (78.5%)
	III - Contaminated	69 (0.9%)	11 (1.3%)	24 (1.9%)	34 (0.6%)
	IV - Dirty/Infected	15 (0.2%)	7 (0.8%)	4 (0.3%)	4 (0.1%)
Operative time (min)		166.00 [122.00 - 219.00]	169.50 [120.75 - 224.25]	169.00 [123.00 - 224.00]	165.00 [123.00 - 217.00]

**GNRI** = geriatric nutritional risk index; BMI = body mass index; ASA = American Society of Anesthesiologists; 5i-mFI = 5-item modified frailty index; COPD = chronic obstructive pulmonary disease; AKI = acute kidney injury

Comparisons of unadjusted 30-day outcomes following nephrectomy among the three study groups were performed (Table-2). Thirty-day outcomes were generally worse for the moderate and severe malnutrition groups compared to patients with a normal nutritional status. The unadjusted 30-day postoperative complication rates were significantly worsened as the severity of malnutrition increased. Overall, malnourished patients (GNRI ≤ 98) had higher rates of a complication compared to patients with a normal nutritional status (GNRI > 98). Likewise, rates of 30-day mortality and major complications (CD III/IV) were significantly higher in malnourished patients compared to those with a normal nutritional status.

**Table 2:** Patient postoperative 30-day outcomes.

Variables	All patients (n=7863)	GNRI <92 (n=872)	GNRI 92-98 (n=1241)	GNRI >98 (n=5750)	p value
Acute Renal Failure	80 (1.0%)	9 (1.0%)	15 (1.2%)	56 (1.0%)	0.8
Any NSQIP Complication	1556 (19.8%)	367 (42.1%)	312 (25.1%)	877 (15.3%)	<0.001
Any Readmission	565 (7.2%)	104 (11.9%)	111 (8.9%)	350 (6.1%)	<0.001
Any Reoperation	196 (2.5%)	27 (3.1%)	34 (2.7%)	135 (2.3%)	0.3
Cardiac Arrest Requiring CPR	43 (0.5%)	7 (0.8%)	12 (1.0%)	24 (0.4%)	0.033
Death (CD V)	61 (0.8%)	18 (2.1%)	15 (1.2%)	28 (0.5%)	<0.001
Deep Incisional SSI	13 (0.2%)	2 (0.2%)	3 (0.2%)	8 (0.1%)	0.6
DVT Requiring Therapy	71 (0.9%)	19 (2.2%)	13 (1.0%)	39 (0.7%)	<0.001
Extended Length of Stay	1529 (19.4%)	322 (36.9%)	304 (24.5%)	903 (15.7%)	<0.001
Major Complications (CD III/IV)	406 (5.2%)	78 (8.9%)	85 (6.8%)	243 (4.2%)	<0.001
Myocardial Infarction	74 (0.9%)	10 (1.1%)	15 (1.2%)	49 (0.9%)	0.4
Non-Home Discharge	581 (7.4%)	165 (18.9%)	131 (10.6%)	285 (5.0%)	<0.001
Organ/Space SSI	54 (0.7%)	10 (1.1%)	8 (0.6%)	36 (0.6%)	0.2
Pneumonia	155 (2.0%)	28 (3.2%)	29 (2.3%)	98 (1.7%)	0.007
Progressive Renal Insufficiency	94 (1.2%)	18 (2.1%)	25 (2.0%)	51 (0.9%)	<0.001
Pulmonary Embolism	53 (0.7%)	15 (1.7%)	11 (0.9%)	27 (0.5%)	<0.001
Sepsis	72 (0.9%)	11 (1.3%)	15 (1.2%)	46 (0.8%)	0.2
Septic Shock	40 (0.5%)	12 (1.4%)	10 (0.8%)	18 (0.3%)	<0.001
Stroke/CVA	26 (0.3%)	4 (0.5%)	3 (0.2%)	19 (0.3%)	0.7
Superficial Incisional SSI	83 (1.1%)	17 (1.9%)	20 (1.6%)	46 (0.8%)	0.001
Transfusions	1039 (13.2%)	290 (33.3%)	207 (16.7%)	542 (9.4%)	<0.001
Unplanned Intubation	104 (1.3%)	25 (2.9%)	25 (2.0%)	54 (0.9%)	<0.001
Urinary Tract Infection	159 (2.0%)	34 (3.9%)	26 (2.1%)	99 (1.7%)	<0.001
Ventilator greater than 48 Hours	84 (1.1%)	24 (2.8%)	15 (1.2%)	45 (0.8%)	<0.001
Wound Disruption	23 (0.3%)	6 (0.7%)	3 (0.2%)	14 (0.2%)	0.072

**GNRI** = geriatric nutritional risk index; **CPR** = cardiopulmonary resuscitation; **CD** = Clavien-Dindo; **SSI** = surgical site infection; **CVA** = cerebrovascular accident

**Figure 3:**
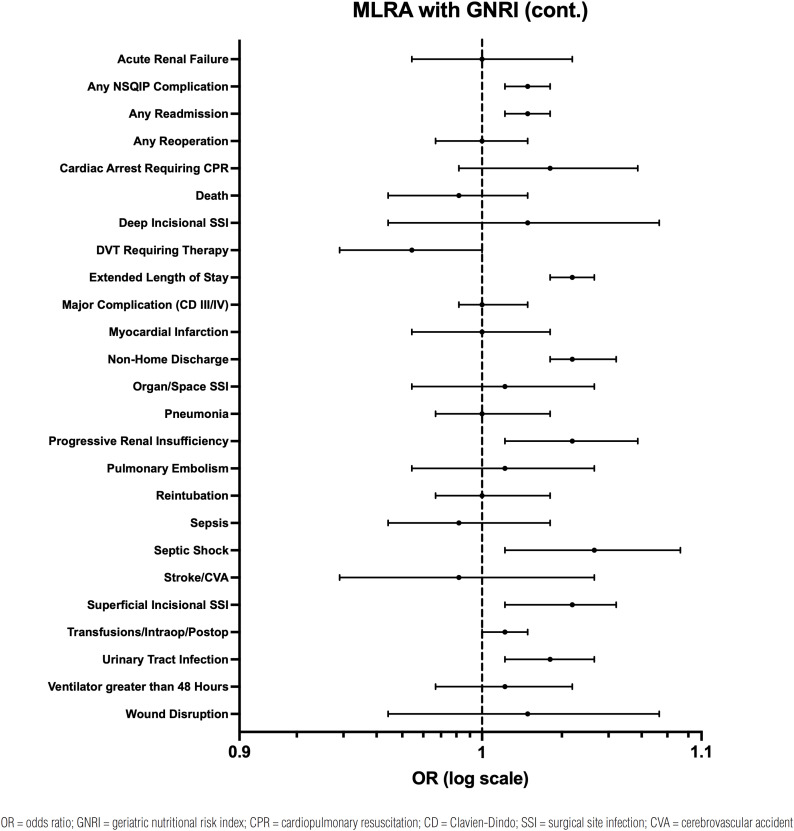
Multivariable logistic regression analysis for complications after adjusting for covariates. GNRI was classified as a continuous variable (per 1 unit decrease).

A MLRA for GNRI defined as a categorical variable was performed (Table-3). After controlling for the confounding variables previously mentioned, malnutrition was found to be independently associated with any readmission (moderate malnutrition: OR 1.30, 95% CI 1.03 – 1.64, p = 0.029; severe malnutrition: OR 1.50, 95% CI 1.14 – 1.98, p = 0.004), progressive renal insufficiency (moderate malnutrition: OR 2.08, 95% CI 1.24 – 3.49, p = 0.006; severe malnutrition: OR 2.03, 95% CI 1.07 – 3.84, p = 0.030), superficial incisional SSI (moderate malnutrition: OR 1.77, 95% CI 1.02 – 3.08, p = 0.044; severe malnutrition: OR 2.04, 95% CI 1.04 – 3.99, p = 0.037), extended LOS (moderate malnutrition: OR 1.50, 95% CI 1.27 – 1.78, p < 0.001; severe malnutrition: OR 2.33, 95% CI 1.91 – 2.85, p < 0.001), and non-home discharge (moderate malnutrition: OR 1.54, 95% CI 1.22 – 1.95, p < 0.001; severe malnutrition: OR 2.34, 95% CI 1.81 – 3.03, p < 0.001). Any NSQIP complication (OR 1.35, 95% CI 1.10 – 1.65, p = 0.004), septic shock (OR 2.93, 95% CI 1.21 – 7.07, p = 0.017), and UTI (OR 2.10, 95% CI 1.31 – 3.35, p = 0.002) were independently associated with severe malnutrition. None of the other postoperative complications were significantly associated with malnutrition. The 30-day outcomes that were independently associated with moderate and severe malnutrition were also statistically significant for the MLRA models using GNRI as a continuous variable (Figure-3). None of the MLRA models had variables with high multicollinearity (VIF ≥ 2.5).

**Table 3:** Multivariable analysis of complications with GNRI as a categorical variable.

Complication	GNRI 92-98		GNRI <92	
	OR [95% CI]	p value	OR [95% CI]	p value
Acute Renal Failure	1.05 [0.56-1.97]	0.9	1.70 [0.75-3.88]	0.20
Any NSQIP Complication	1.10 [0.93-1.31]	0.3	1.35 [1.10-1.65]	**0.004**
Any Readmission	1.30 [1.03-1.64]	**0.029**	1.50 [1.14-1.98]	**0.004**
Any Reoperation	1.06 [0.71-1.58]	0.8	1.09 [0.67-1.77]	0.7
Cardiac Arrest Requiring CPR	0.59 [0.28-1.23]	0.16	1.07 [0.40-2.81]	0.9
Death (CD V)	1.46 [0.75-2.85]	0.3	1.27 [0.61-2.65]	0.5
Deep Incisional SSI	0.65 [0.16-2.61]	0.5	0.86 [0.12-6.08]	0.9
DVT Requiring Therapy	0.97 [0.49-1.90]	0.9	0.60 [0.30-1.20]	0.15
Extended Length of Stay	1.50 [1.27-1.78]	**<0.001**	2.33 [1.91-2.85]	**<0.001**
Major Complication (CD III/IV)	1.17 [0.89-1.54]	0.3	1.09 [0.79-1.52]	0.6
Myocardial Infarction	0.96 [0.52-1.77]	0.9	1.31 [0.60-2.86]	0.5
Non-Home Discharge	1.54 [1.22-1.95]	**<0.001**	2.34 [1.81-3.03]	**<0.001**
Organ/Space SSI	0.89 [0.40-1.99]	0.8	1.29 [0.55-3.03]	0.6
Pneumonia	0.91 [0.58-1.42]	0.7	0.89 [0.53-1.48]	0.6
Progressive Renal Insufficiency	2.08 [1.24-3.49]	**0.006**	2.03 [1.07-3.84]	**0.030**
Pulmonary Embolism	1.37 [0.65-2.91]	0.4	1.89 [0.85-4.23]	0.12
Reintubation	1.42 [0.85-2.37]	0.18	1.30 [0.72-2.35]	0.4
Sepsis	1.12 [0.61-2.08]	0.7	0.84 [0.39-1.82]	0.7
Septic Shock	1.84 [0.80-4.25]	0.15	2.93 [1.21-7.07]	**0.017**
Stroke/CVA	0.55 [0.15-1.93]	0.3	0.75 [0.21-2.66]	0.7
Superficial Incisional SSI	1.77 [1.02-3.08]	**0.044**	2.04 [1.04-3.99]	**0.037**
Transfusions/Intraop/Postop	0.94 [0.75-1.16]	0.5	1.17 [0.93-1.48]	0.18
Urinary Tract Infection	1.10 [0.70-1.73]	0.7	2.10 [1.31-3.35]	**0.002**
Ventilator greater than 48 Hours	0.78 [0.40-1.52]	0.5	1.23 [0.65-2.34]	0.5
Wound Disruption	0.77 [0.21-2.87]	0.7	2.13 [0.70-6.52]	0.19

GNRI as a categorical variable with the reference category as “Normal GNRI”.

OR = odds ratio; CI = confidence interval; GNRI = geriatric nutritional risk index; NSQIP = National Surgical Quality Improvement Program; CPR = cardiopulmonary resuscitation; CD = Clavien-Dindo; SSI = surgical site infection; CVA - cerebrovascular accident OR = odds ratio; GNRI = geriatric nutritional risk index; CPR = cardiopulmonary resuscitation; CD = Clavien-Dindo; SSI = surgical site infection; CVA = cerebrovascular accident

A sensitivity analysis was performed after excluding 513 (6.5%) patients who had disseminated cancer at the time of nephrectomy (Supplementary Table-1). Progressive renal insufficiency, extended LOS, and non-home discharge remained independently associated with moderate and severe malnutrition. Likewise, septic shock and UTI continued to be independently associated with severe malnutrition. Readmission was independently associated with severe malnutrition only. Superficial incisional SSI was not independently associated with either moderate or severe malnutrition.

## DISCUSSION

Complications after nephrectomy place a significant burden on both the oncological patient and the health care system. The use of predictors for adverse postoperative outcomes is crucial in value-based healthcare as it can aid in better resource management. Although preoperative malnutrition is a well-recognized predictor of poor outcomes following major oncologic surgery, the role of GNRI in evaluating malnutrition and its association with 30-day outcomes following nephrectomy for renal cancer has not been thoroughly studied before (5, 16). As defined by a GNRI ≤ 98, the prevalence of malnutrition in our cohort was 27.3%, which falls within the range of previously reported data (6).

Using MLRA, we found that GNRI was independently associated with readmission, progressive renal insufficiency, superficial incisional SSI, septic shock, UTI, extended LOS, and non-home discharge. After excluding patients with disseminated cancer, the sensitivity analysis yielded similar results with the exceptions of readmission and superficial incisional SSI no longer being independently associated with either moderate or severe malnutrition. This data suggests that malnutrition impacts on 30-day outcomes after nephrectomy, regardless of metastatic disease. The study by May et al. using NSQIP to describe rates of 30-day complications after cytoreductive nephrectomy found malnutrition is not an independent predictor of severe or overall postoperative morbidity; however, their definition was based on a BMI < 18.5 kg/m² (24).

Malnutrition is not only associated with poor postoperative outcomes, but also worse long-term survival, quality of life, and chemotherapy toxicity among oncology patients (25, 26). Expert consensus recommends perioperative nutrition screening in elderly patients with the Mini Nutritional Assessment Short Form (MNA-SF) (27). However, this assessment utilizes questions that rely on patient-reported data which translates to poor interobserver reliability. The MNA-SF also requires training to administer, and is not readily available once the patient arrives to the hospital (28). In contrast, the GNRI relies on objective data that could be easily accessible for most patients perioperatively (11). The GNRI also employs a standardized formula that does not require training and could potentially be administered by any health care team member. Gu et al. found that the GNRI was superior to the MNA-SF for risk discrimination regarding overall mortality in patients with renal cancer (14). Other studies have found the GNRI to be an independent predictor of both short and long-term outcomes following nephrectomy for renal cancer (15, 16).

Although the present study uses a large population to evaluate GNRI as a predictor of complications following nephrectomy, it has inherent limitations that apply to the usage of large clinical databases. First, the NSQIP database limits follow-up to 30 days after the procedure of interest; thus, complications that occur either past 30 days or long-term cannot be analyzed. Second, the cohort is limited to patients with complete anthropometric data and serum albumin measured within 30 days prior to nephrectomy; this excludes more than 10,000 patients from being potentially included in our study. Third, the general NSQIP file does not collect data on TNM staging, histologic features, or adjuvant therapy, which are prognostic factors that influence outcomes in patients with renal cancer (3, 29, 30). Fourth, NSQIP does not have data on nephrectomy-specific complications which hinders a more granular analysis of postoperative complications. Our findings could be more accurately evaluated in prospective studies, which should systematically include the assessment of the missing variables in question.

## CONCLUSIONS

In the setting of nephrectomy for renal cancer, a GNRI ≤ 98 is an independent predictor of 30-day readmission, progressive renal insufficiency, superficial incisional SSI, septic shock, UTI, extended LOS, and non-home discharge. GNRI could be used to assess nutritional status in elderly patients with renal cancer and counsel them prior to nephrectomy.

## ABBREVIATIONS

MI: minimally invasive
GNRI: Geriatric Nutritional Risk Index
ACS-NSQIP: American College of Surgeons National Surgical Quality Improvement Program
ICD: International Coding of Diseases
CPT: Current Procedural Terminology
ASA: American Society of Anesthesiology
BMI: body mass index
5i-Mfi: 5-item modified frailty index
DM: diabetes mellitus
HT: hypertension
CHF: congestive heart failure
COPD: chronic obstructive pulmonary disease
CBW: current body weight
IBW: ideal body weight
PE: pulmonary embolism
DVT: deep venous thrombosis
UTI: urinary tract infection
SSI: surgical site infection
CD: Clavien-Dindo
LOS: length of stay
IQR: interquartile range
MLRA: Multivariable logistic regression analysis

## References

[1] Sung H, Ferlay J, Siegel RL, Laversanne M, Soerjomataram I, Jemal A (2021). Global Cancer Statistics 2020: GLOBOCAN Estimates of Incidence and Mortality Worldwide for 36 Cancers in 185 Countries. CA Cancer J Clin.

[2] Kanesvaran R, Le Saux O, Motzer R, Choueiri TK, Scotté F, Bellmunt J (2018). Elderly patients with metastatic renal cell carcinoma: position paper from the International Society of Geriatric Oncology. Lancet Oncol.

[3] Motzer RJ, Jonasch E, Boyle S, Carlo MI, Manley B, Agarwal N (2020). NCCN Guidelines Insights: Kidney Cancer, Version 1.2021. J Natl Compr Canc Netw.

[4] MacLennan S, Imamura M, Lapitan MC, Omar MI, Lam TB, Hilvano-Cabungcal AM (2012). Systematic review of perioperative and quality-of-life outcomes following surgical management of localised renal cancer. Eur Urol.

[5] van Stijn MF, Korkic-Halilovic I, Bakker MS, van der Ploeg T, van Leeuwen PA, Houdijk AP (2013). Preoperative nutrition status and postoperative outcome in elderly general surgery patients: a systematic review. JPEN J Parenter Enteral Nutr.

[6] D’Almeida CA, Peres WAF, de Pinho NB, Martucci RB, Rodrigues VD, Ramalho A (2020). Prevalence of Malnutrition in Older Hospitalized Cancer Patients: A Multicenter and Multiregional Study. J Nutr Health Aging.

[7] Soeters PB, Reijven PL, van Bokhorst-de van der Schueren MA, Schols JM, Halfens RJ, Meijers JM (2008). A rational approach to nutritional assessment. Clin Nutr.

[8] Paillaud E, Caillet P, Campillo B, Bories PN (2006). Increased risk of alteration of nutritional status in hospitalized elderly patients with advanced cancer. J Nutr Health Aging.

[9] Langley-Evans SC (2021). Nutrition screening tools: Still no consensus 40 years on. J Hum Nutr Diet.

[10] van Bokhorst-de van der Schueren MA, Guaitoli PR, Jansma EP, de Vet HC (2014). Nutrition screening tools: does one size fit all? A systematic review of screening tools for the hospital setting. Clin Nutr.

[11] Bouillanne O, Morineau G, Dupont C, Coulombel I, Vincent JP, Nicolis I (2005). Geriatric Nutritional Risk Index: a new index for evaluating at-risk elderly medical patients. Am J Clin Nutr.

[12] Lidoriki I, Schizas D, Frountzas M, Machairas N, Prodromidou A, Kapelouzou A (2021). GNRI as a Prognostic Factor for Outcomes in Cancer Patients: A Systematic Review of the Literature. Nutr Cancer.

[13] Xie H, Tang S, Wei L, Gan J (2020). Geriatric nutritional risk index as a predictor of complications and long-term outcomes in patients with gastrointestinal malignancy: a systematic review and meta-analysis. Cancer Cell Int.

[14] Gu W, Zhang G, Sun L, Ma Q, Cheng Y, Zhang H (2015). Nutritional screening is strongly associated with overall survival in patients treated with targeted agents for metastatic renal cell carcinoma. J Cachexia Sarcopenia Muscle.

[15] Kang HW, Seo SP, Kim WT, Yun SJ, Lee SC, Kim WJ (2020). A Low Geriatric Nutritional Risk Index is Associated with Aggressive Pathologic Characteristics and Poor Survival after Nephrectomy in Clear Renal Cell Carcinoma: A Multicenter Retrospective Study. Nutr Cancer.

[16] Watanabe D, Miura K, Yamashita A, Minowa T, Uehara Y, Mizushima S (2021). A Comparison of the Predictive Role of the Geriatric Nutritional Risk Index and Immunonutritional Parameters for Postoperative Complications in Elderly Patients with Renal Cell Carcinoma. J Invest Surg.

[17] American College of Surgeons (2019). ACS NSQIP Operations Manual: Chapter 4 - Variables and Definitions.

[18] Goldwag J, Harris A, Bettis AD (2021). 5-Item Modified Frailty Index as a Preoperative Predictor of Morbidity Following Minimally Invasive Partial Nephrectomy. Urology.

[19] Taylor BL, Xia L, Guzzo TJ, Scherr DS, Hu JC (2019). Frailty and Greater Health Care Resource Utilization Following Major Urologic Oncology Surgery. Eur Urol Oncol.

[20] Subramaniam S, Aalberg JJ, Soriano RP, Divino CM (2018). New 5-Factor Modified Frailty Index Using American College of Surgeons NSQIP Data. J Am Coll Surg.

[21] Lemmens HJ, Brodsky JB, Bernstein DP (2005). Estimating ideal body weight--a new formula. Obes Surg.

[22] Dindo D, Demartines N, Clavien PA (2004). Classification of surgical complications: a new proposal with evaluation in a cohort of 6336 patients and results of a survey. Ann Surg.

[23] Kanda Y (2013). Investigation of the freely available easy-to-use software ‘EZR’ for medical statistics. Bone Marrow Transplant.

[24] May DN, Hill H, Matrana MR, Lata-Arias K, Canter DJ (2021). A Contemporary Analysis of the 30-day Morbidity and Mortality Associated With Cytoreductive Nephrectomy. Urology.

[25] Bozzetti F (2015). Why the oncologist should consider the nutritional status of the elderly cancer patient. Nutrition.

[26] Wu J, Yang N, Yuan M (2021). Dietary and circulating vitamin D and risk of renal cell carcinoma: a meta-analysis of observational studies. Int Braz J Urol.

[27] Lobo DN, Gianotti L, Adiamah A, Barazzoni R, Deutz NEP, Dhatariya K (2020). Perioperative nutrition: Recommendations from the ESPEN expert group. Clin Nutr.

[28] Trampisch US, Pourhassan M, Daubert D, Volkert D, Wirth R (2022). Interrater reliability of routine screening for risk of malnutrition with the Mini Nutritional Assessment Short-Form in hospital. Eur J Clin Nutr.

[29] Alvim R, Tin A, Nogueira L, Lebdai S, Wong N, Takeda T (2021). A comparison of oncologic and functional outcomes in patients with pt3a renal cell carcinoma treated with partial and radical nephrectomy. Int Braz J Urol.

[30] Wang K, Wu Z, Wang G, Shi H, Xie J, Yin L (2021). Survival nomogram for patients with bone metastatic renal cell carcinoma: A population-based study. Int Braz J Urol.

